# MUTC-PD-Based High-Efficiency Photonic Terahertz Generation and Radiation in the 275–296 GHz Band

**DOI:** 10.3390/mi17020196

**Published:** 2026-01-31

**Authors:** Yun Wang, Xiaorui Liu, Jianguo Yu

**Affiliations:** School of Electronic Engineering, Beijing University of Posts and Telecommunications, Beijing 100876, China; wyun@bupt.edu.cn (Y.W.); lxr1100@bupt.edu.cn (X.L.)

**Keywords:** MUTC-PD, impedance matching, power combiner, photonic antenna

## Abstract

We design a photonic terahertz (THz) antenna operating in the 275–296 GHz band, integrating two identical modified uni-traveling-carrier photodiodes (MUTC-PDs), two impedance matching networks, a Wilkinson power combiner and a Vivaldi antenna. Simulation results show a saturated output power of 1.58 dBm at 280 GHz, achieving a 4.13× power enhancement through optimized impedance and power combining compared to a standalone device. The integrated antenna achieves a peak gain of 7.93 dBi, with reflection coefficients below −10 dB. The system demonstrates a power combining efficiency exceeding 95% for phase imbalances up to 20°, and the Wilkinson combiner exhibits only 0.76 dB insertion loss at 285 GHz, demonstrating high radiation and combining efficiency in the 275–296 GHz band.

## 1. Introduction

Building upon our previous study on bandwidth-enhanced MUTC-PDs [1], this work further integrates passive and radiating components to realize a compact photonic THz antenna. The terahertz (THz, 0.1–10 THz) frequency range has emerged as a promising frontier for next-generation communication systems, offering unparalleled bandwidth, low latency, and high spatial resolution [2,3,4,5,6]. Particularly, the sub-range spanning 275–450 GHz is gaining significant attention as a key resource for future 6G wireless networks and ultra-high-resolution applications, owing to the availability of vast contiguous bandwidth capable of supporting terabit-per-second data rates and millimeter-scale imaging resolution. Recent experimental breakthroughs in this frequency window have successfully demonstrated 3D terahertz imaging utilizing chromatic metasurfaces, providing a powerful tool for high-precision spatial detection [7]. Beyond 6G communications, this band has also exhibited immense potential for non-contact sensing, such as the real-time quantification of plant leaf water status for sustainable agriculture [8]. To fully exploit these capabilities, ongoing research continues to explore broadband wave manipulation through advanced metasurface designs based on circuit-model optimization, facilitating more versatile THz systems [9].

The uni-traveling-carrier photodiode (UTC-PD), known for its wide bandwidth, fast response time, and high output power, plays a pivotal role in THz signal generation and photonic communication systems [10,11]. The modified UTC-PD (MUTC-PD) further enhances responsivity without compromising bandwidth; however, its output power remains limited beyond 200 GHz. To address this limitation, integrating MUTC-PDs with impedance-matching networks, power combiners, and broadband antennas is crucial for improving efficiency at higher frequencies.

While electronic THz emitters such as resonant tunneling diodes (RTDs) have achieved notable progress in compact source design, their output power and conversion efficiency rapidly deteriorate at higher frequencies [12,13]. Recent studies have demonstrated oscillation frequencies beyond 2 THz and milliwatt-level power at 1 THz [14], yet the DC-to-RF efficiency remains below 1%, and parasitic resistance, carrier transit-time delay, and thermal limitations restrict scalability [15]. Moreover, RTD oscillators typically require resonant cavities for impedance matching, complicating integration with photonic platforms. In contrast, photomixing emitters based on UTC-PDs offer broadband, tunable, and phase-stable THz generation without electronic oscillation.

Several integrated approaches have been reported to enhance the efficiency of photonic THz emitters. Song et al. demonstrated a WR3 waveguide module integrating two UTC-PDs with a T-junction power combiner, achieving 1.2 mW at 300 GHz [16]. Sun et al. realized an MUTC-PD integrated with a Vivaldi antenna and matching network, yielding 5 dBm EIRP at 100 GHz [17]. Ohara et al. and Nagatsuma et al. further developed integrated UTC-PD emitters, achieving >2 mW at 300 GHz and 200 Gbit/s wireless transmission [18,19]. Ssali et al. provided a scalable solution for THz wave generation by integrating a 4 × 1 UTC-PD array with a T-junction combiner and antenna, achieving a 10.9 dB increase in detected power at 300 GHz [20]. Despite these achievements, issues such as low radiation efficiency and fabrication complexity persist, particularly for SiC substrates with high dielectric constants.

To achieve efficient terahertz emission and lay the groundwork for future broadband systems, we design a photonic terahertz (THz) antenna operating within the 275–296 GHz band. This system integrates two identical modified uni-traveling-carrier photodiodes (MUTC-PDs), two impedance matching networks, a Wilkinson power combiner, and a Vivaldi antenna. This frequency band serves as a critical stepping stone towards the 275–450 GHz range, allowing for the validation of key device optimizations and integration concepts under current technological constraints. Initial experimental testing revealed that a first-generation MUTC-PD with a 130 nm depletion region suffered from velocity overshoot failure, which underscored the necessity of optimizing both the epitaxial structure and contact resistance. Informed by these findings, we refined the device design and performed full-system integration and simulation. The simulated results show a saturated output power of 1.58 dBm at 280 GHz, corresponding to a 4.13× power enhancement through efficient impedance matching and power combining compared to a standalone device. The integrated antenna exhibits a peak gain of 7.93 dBi and maintains reflection coefficients below −10 dB across the operating band.

## 2. Modeling and Analysis of the MUTC-PD

### 2.1. Design of the Epitaxial Structure

The epitaxial design of MUTC-PD focuses on minimizing carrier transit time and the RC time constant to enhance high-frequency performance. Carrier transit time is influenced by diffusion, drift, and velocity overshoot effects, determined by the thicknesses of the undepleted absorption layer (*W_a_*), depleted absorption layer (*W_ad_*), and collection layer (*W_c_*), respectively [21]. The RC time constant is mainly governed by the junction area, collection layer thickness, and the contact resistance of the P- and N-type electrodes.

**Figure 1:**
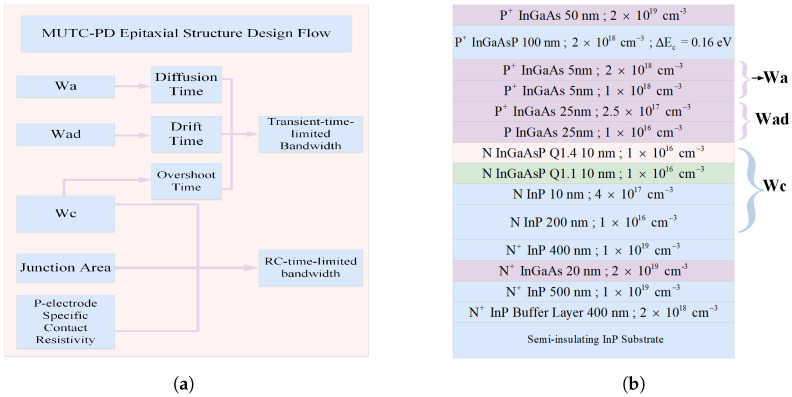
(**a**) Schematic workflow and (**b**) corresponding epitaxial layer configuration of the proposed MUTC-PD. In (**b**), each layer is labeled with its thickness (nm) and carrier doping concentration (cm^−3^), separated by a semicolon (e.g., 50 nm; 2 × 10^19^ cm^−3^).

To optimize performance, the absorption layer thickness must be carefully balanced to ensure sufficient optical absorption while minimizing diffusion and drift delays. The collection layer thickness influences the junction capacitance. The electric field in the depletion region, which controls velocity overshoot [22], is adjusted by modifying the doping concentration in the cliff layer and the conduction band offset of the barrier material relative to InGaAs. Additionally, minimizing the junction area and contact resistance helps reduce the RC-limited bandwidth. The MUTC-PD design flow is illustrated in Figure 1a, and the corresponding epitaxial structure is shown in Figure 1b.

**Figure 2:**
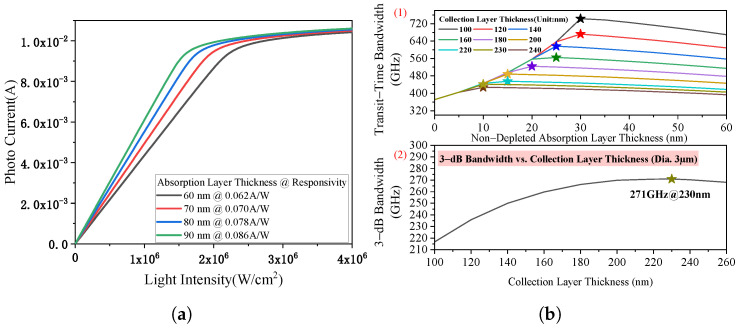
(**a**) Simulated responsivity dependence on absorption layer thicknesses and injection light intensity. A thickness of 60 nm yields a responsivity of 0.062 A/W, balancing bandwidth and optical-to-electrical conversion efficiency. (**b**) Analysis of bandwidth performance: (1) transit-time-limited bandwidth as a function of non−depleted absorption layer thickness, with the collection layer thickness varying from 100 nm to 240 nm; (2) 3-dB bandwidth as a function of collection layer thickness. The asterisk (*) indicates the maximum 3-dB bandwidth of 271 GHz achieved at a collection layer thickness of 230 nm.

To achieve an intrinsic 3−dB bandwidth exceeding 100 GHz, the absorption layer must be reduced in thickness. As shown in Figure 2a, the responsivity decreases as the absorption layer becomes thinner, reaching a minimum of 0.062 A/W at 60 nm. Reducing the thickness further causes the responsivity to drop below 0.05 A/W, so a thickness of 60 nm is selected as the optimal balance between bandwidth and responsivity. The overall 3−dB bandwidth is jointly determined by carrier transit time and RC limitations. Simulation results in Figure 2b show that a maximum 3−dB bandwidth of 271 GHz can be achieved with a 10 nm undepleted absorption layer and a 230 nm collection layer, with the active region diameter set to 3 μm.

**Figure 3:**
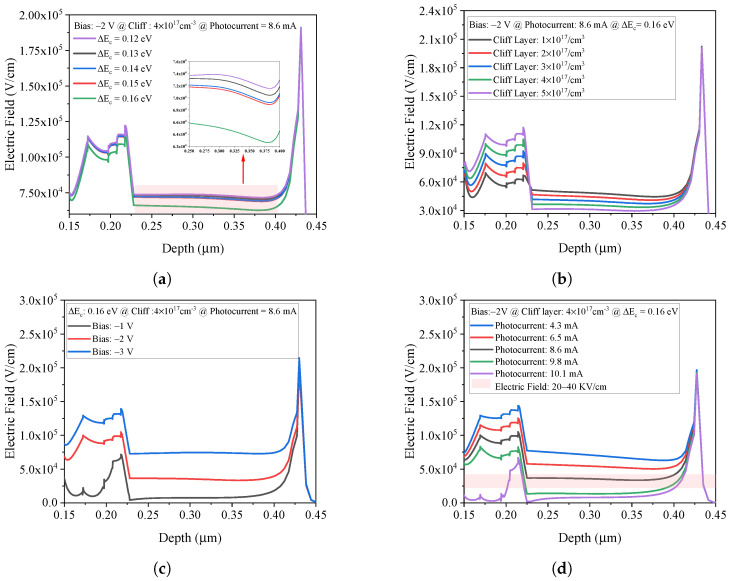
(**a**) Electric field in the depletion region as a function of the conduction band offset (Δ*E_c_*), with larger Δ*E_c_* leading to a lower electric field in the collection region under fixed reverse bias and incident light intensity. (**b**) Electric field versus cliff layer doping concentration at a fixed Δ*E_c_*, where increasing doping concentration results in a lower electric field in the collection region under fixed reverse bias and light intensity. (**c**) Electric field profile across the intrinsic region under different reverse bias voltages, showing that increasing reverse bias increases the electric field in the collection region. (**d**) Electric field in the collection region versus output photocurrent, highlighting the velocity overshoot window (20−40 kV/cm).

**Figure 4:**
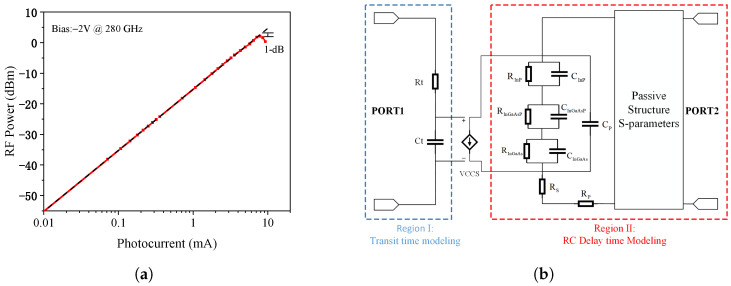
(**a**) RF power versus photocurrent at 280 GHz. (**b**) Detailed equivalent-circuit model of the MUTC-PD including transit-time and RC delay submodules.

Velocity overshoot in the collection region is critical for achieving this bandwidth, requiring the electric field to be maintained within 20–40 kV/cm [23,24]. As shown in Figure 3a–d, the field distribution is influenced by the conduction band offset (Δ*E_c_*), cliff layer doping concentration, and applied bias. With optimized conditions—Δ*E_c_* = 0.16 eV, doping concentration of 4 × 10^17^ cm^−3^, and a reverse bias of −2 V—the electric field remains within the optimal range up to a photocurrent of 8.6 mA. Beyond this point, space-charge effects cause the electric field to collapse. Based on this analysis, Figure 4a presents the simulated output power as a function of photocurrent at 280 GHz. The discrete MUTC-PD reaches a saturated output power of 1.58 dBm at a photocurrent of 7.86 mA.

### 2.2. Accurate Modeling of the MUTC-PD

In the previous section, a simplified model was adopted [25]; the equivalent resistance of the MUTC-PD included only the contact resistances of the P- and N-type electrodes and the load resistance, assuming a specific contact resistivity of 1 × 10^−6^ Ω · cm^2^ for the P electrode. However, this approach lacks the accuracy required to reflect the actual behavior of the device. A more refined model must account for the junction capacitances and series resistances of both the collection region and the depleted absorption region. In addition, the equivalent resistance and capacitance of the InGaAsP (Q1.4) and InGaAsP (Q1.1) buffer layers must also be considered [26].

Figure 4b illustrates the detailed equivalent circuit model of the MUTC-PD, which consists of two main parts: the transit-time model and the RC delay model. In this model, the injected photocurrent is represented by an ideal voltage-controlled current source. Region 1 comprises a series resistance $R_{t}$ and a capacitance $C_{t}$, where the voltage across $C_{t}$ governs the operating state of Region 2. The values of $R_{t}$ and $C_{t}$ are determined by the transit-time bandwidth. Region 2 includes not only the equivalent resistances and capacitances of individual layers but also parasitic resistances and capacitances, as well as the series resistances introduced by the P and N electrodes. Furthermore, the S-parameters of passive circuits, as discussed in the next section, are also incorporated into the model.

Table 1 lists the specific parameter values used in the equivalent circuit, with their derivations detailed in our previous work [1]. Using the parameters from Table 1, the intrinsic bandwidth is calculated to be 124 GHz.

## 3. Design of Passive Components

### 3.1. Design of Photonic Terahertz Generator

As demonstrated in the previous section, even reducing the absorption layer thickness to 60 nm fails to significantly improve the frequency response of the MUTC-PD beyond 200 GHz without applying bandwidth enhancement techniques such as inductive peaking at the output electrode. To enable photonic antenna operation in the 275–296 GHz band, improving the MUTC-PD’s output power within this frequency band is crucial.

**Figure 5:**
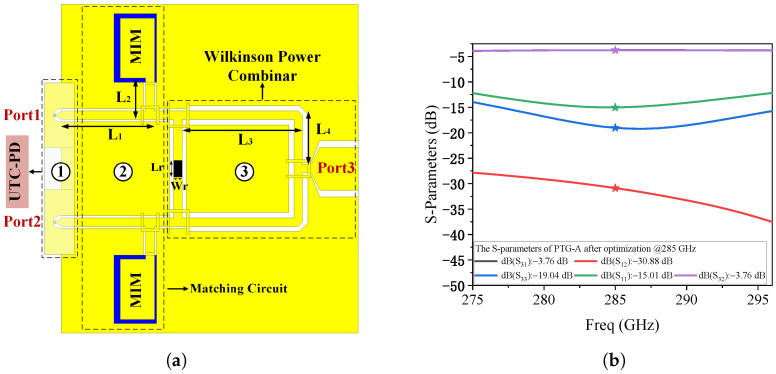
(**a**) Schematic top view of the proposed Photonic Terahertz Generator-A (PTG-A). (**b**) Simulated S-parameters of PTG-A.

In this work, we propose a passive integration approach—designated as the Photonic Terahertz Generator-A (PTG-A)—which incorporates an impedance matching circuit and a Wilkinson power combiner to address this challenge. The impedance matching circuit is optimized to match the MUTC-PD’s output impedance to 50 Ω at 285 GHz [27]. The Wilkinson power combiner, comprising three well-matched ports, is employed to coherently combine the outputs of two identical MUTC-PDs. A schematic top view of PTG-A is shown in Figure 5a. The matching network consists of a series coplanar waveguide (CPW) of length $L_{1}$, followed by a short-circuited CPW of length $L_{2}$, whose terminal is connected to the top electrode of a metal–insulator–metal (MIM) capacitor functioning as a DC block.

The Wilkinson power combiner in PTG-A employs CPWs with a characteristic impedance of 100 Ω at its input ports [28,29]. The isolation resistor is implemented using a material with an aspect ratio ($L_{r}$/$W_{r}$) of 4, with its thickness and conductivity carefully optimized to achieve a resistance of 200 Ω. Detailed dimensional parameters are provided in Table 2. All passive components are designed on a quartz substrate ($\epsilon_{r}$ = 3.78). The MUTC-PD, which is fabricated on an InP substrate, is flip-chip bonded onto the quartz-based passive circuit. This hybrid integration approach not only ensures reliable high-frequency performance but also significantly lowers the overall manufacturing cost, making it suitable for scalable production.

The simulated performance of PTG-A, obtained using High Frequency Structure Simulator (HFSS), is presented in Figure 5b. The transmission loss from the two input ports to the output port is approximately 3.76 dB, indicating that the actual loss, after accounting for the ideal 3 dB power split, is only about 0.76 dB. The reflection coefficients at ports 1 and 2 remain below −10 dB across the 275–296 GHz band, and reach a minimum of −15.01 dB at 285 GHz. Furthermore, the isolation between the two input ports is below −25 dB, indicating excellent port isolation and minimal crosstalk.

**Figure 6:**
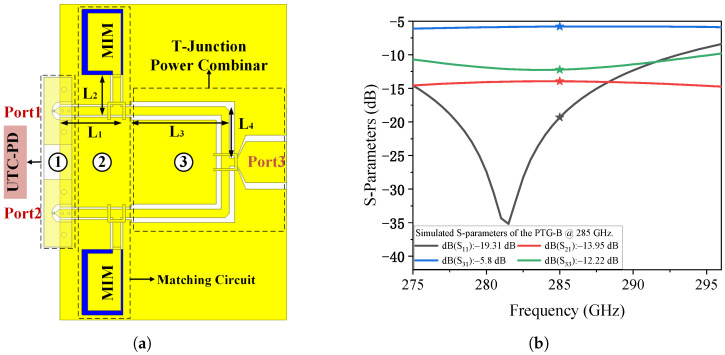
(**a**) Schematic top view of the proposed Photonic Terahertz Generator-B (PTG-B). (**b**) Simulated S-parameters of PTG-B.

For comparison, the top-view schematic of PTG-B is shown in Figure 6a. PTG-B replaces the Wilkinson combiner with a traditional T-junction power combiner. As shown in Figure 6b, this change leads to a degradation in performance. The transmission loss from the two input ports to the output port is approximately 5.8 dB, indicating that the actual loss, after accounting for the ideal 3 dB power split, is about 2.8 dB. The reflection coefficients at ports 1 and 2 are approximately −19.31 dB, and the isolation between the two ports is around −13.95 dB. These results demonstrate that the Wilkinson-based PTG-A offers significantly improved impedance matching and isolation characteristics compared to the T-junction implementation, thereby providing a more effective solution for high-frequency photonic THz signal generation.

**Figure 7:**
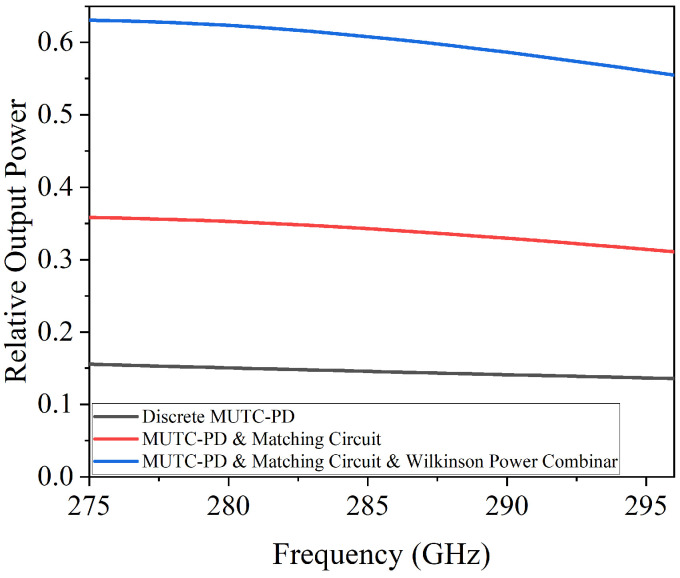
Relative output power across three configurations: a discrete MUTC-PD, an MUTC-PD with only the impedance matching circuit, and PTG-A.

By importing the S-parameters of PTG-A into the equivalent circuit model shown in Figure 4b—where ports 1 and 2 are connected to the outputs of two identical MUTC-PDs, and port 3 is terminated with a 50 Ω load—we compared the relative output power across three configurations in Figure 7: a discrete MUTC-PD, an MUTC-PD with only the impedance matching circuit, and PTG-A. The results reveal that incorporating the impedance matching circuit significantly enhances the output power at the resonant frequency. Specifically, at 280 GHz, the relative output power increases from 0.15 (single MUTC-PD) to 0.352. However, this improvement is still limited. When the Wilkinson power combiner is added to PTG-A, the relative output power at 280 GHz rises to 0.623, an increase of 4.13 times compared to the single MUTC-PD. Building upon the PTG-A configuration that achieves optimized impedance and power combining, a broadband radiating structure is subsequently designed to realize free-space THz emission.

### 3.2. Fabrication Tolerance Analysis

To rigorously address the fabrication challenges at terahertz frequencies, a systematic tolerance analysis was performed for the integrated PTG-A. We evaluated the sensitivity of the S-parameters ($S_{11}$, $S_{21}$, $S_{31}$, and $S_{33}$) by introducing deliberate dimensional deviations to three critical geometric parameters: the CPW gap width, the signal line width, and the MIM capacitor width.

**Figure 8:**
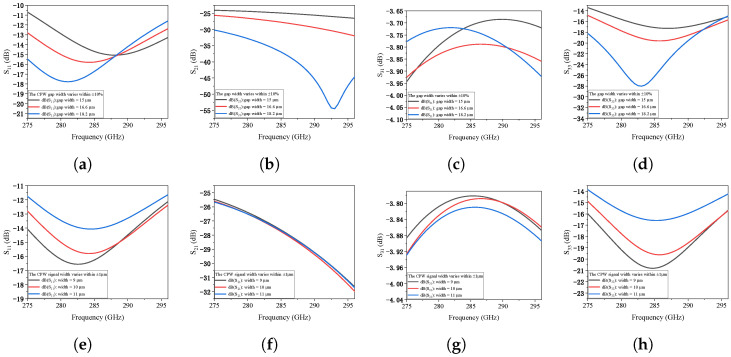
Fabrication tolerance analysis of the proposed PTG-A S-parameters under CPW dimensional variations: (**a**–**d**) ±10% deviation in CPW gap width, and (**e**–**h**) ±1 μm deviation in CPW signal line width.

**Figure 9:**
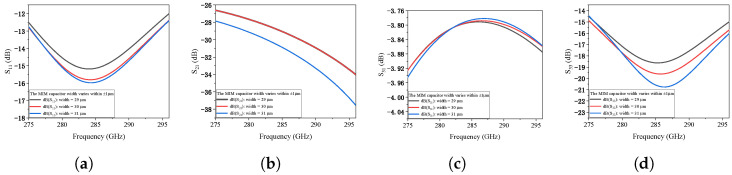
Fabrication tolerance analysis of the proposed PTG-A S-parameters under MIM capacitor width variations: (**a**–**d**) ±1 μm deviation in MIM capacitor width.

**Figure 10:**
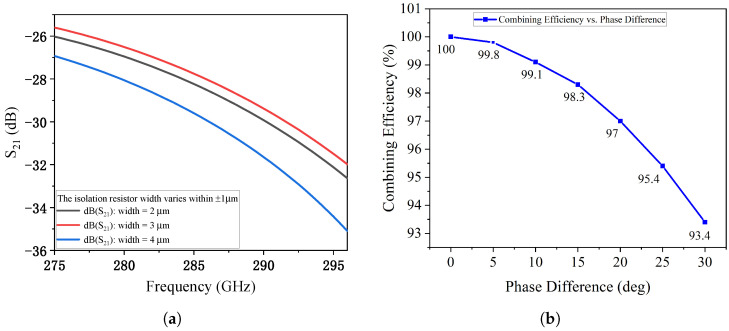
Further sensitivity analysis of the PTG-A: (**a**) Isolation between Port 1 and Port 2 with respect to isolation resistor width variations. (**b**) Power combining efficiency as a function of phase mismatch between the two MUTC-PDs.

The fabrication robustness of the proposed PTG-A was further investigated by introducing dimensional deviations. When the gap width between the signal line and ground plane is varied by ±10%, the impedance matching remains stable. As shown in Figure 8a–d, both input reflection ($S_{11}$) and output reflection ($S_{33}$) stay well below −10 dB, confirming the design’s resilience to over-etching or under-etching. Figure 8e–h shows the impact of a ±1 μm deviation in the signal line width. The transmission coefficients $S_{21}$ and $S_{31}$ are nearly identical to the optimized case, ensuring stable power combining efficiency. Furthermore, the sensitivity to MIM capacitor dimensions was evaluated in Figure 9. Adjusting the MIM capacitor width by ±1 μm results in negligible frequency shifts and magnitude variations as depicted in Figure 9a–d. Additionally, the influence of the isolation resistor width on the port-to-port isolation was analyzed. As shown in Figure 10a, the isolation between Port 1 and Port 2 remains better than 20 dB across the target band despite width variations, ensuring effective decoupling between the two MUTC-PD sources. Finally, considering potential synchronization errors in the optical feeding network, the combining efficiency as a function of phase mismatch between the two PDs was investigated. Figure 10b demonstrates that the PTG-A maintains a combining efficiency of over 95% for phase imbalances up to 20°, further validating the robustness of the proposed architecture. These results confirm that the proposed PTG-A possesses a generous design margin. The stability under these variations significantly relaxes the fabrication precision requirement from the initially estimated 0.5 μm to a more practical range, ensuring a high manufacturing yield.

## 4. Design of Photonic Antenna

### 4.1. Design of Vivaldi Antenna

**Figure 11:**
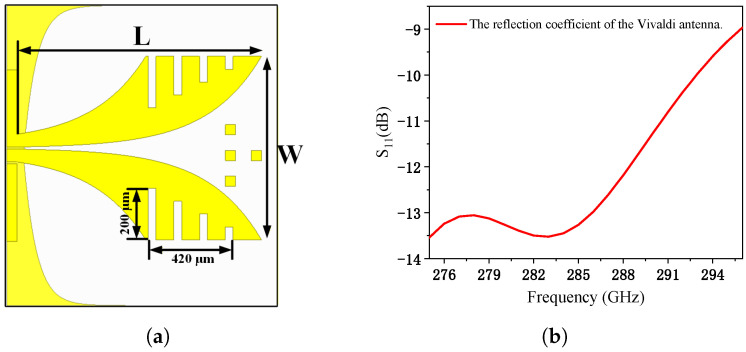
(**a**) Schematic diagram of the Vivaldi antenna; (**b**) simulated reflection coefficient across 275–296 GHz.

To achieve efficient radiation and broad bandwidth in high-frequency applications, the Vivaldi antenna is often chosen due to its wideband performance and compact design [30,31]. The Vivaldi antenna consists of a feed port, a radiating structure, and a reflecting structure on the backside. The feed port uses a coplanar waveguide, matching the third port of the Wilkinson power combiner. The schematic diagram of the Vivaldi antenna is shown in Figure 11a, and its reflection performance is illustrated in Figure 11b. The radiating structure features exponentially tapered slots designed for broadband radiation. To further enhance gain, multiple slots are introduced into the radiating element, and several metal blocks are embedded on the center of the dielectric substrate to improve signal guidance. Each slot is 30 μm wide, with a spacing of 100 μm between adjacent slots. The slot height starts at 200 μm and decreases stepwise by 50 μm along the taper.

On the backside of the dielectric substrate, an arc-shaped reflecting structure is designed to suppress backlobe reflection. For operation within the 275–296 GHz frequency range, the antenna’s physical dimensions are optimized to meet efficient radiation conditions. Specifically, the maximum aperture must be at least half the wavelength at the lowest frequency (275 GHz), which corresponds to approximately 0.55 mm. The length of the exponential taper section, L, is chosen as 1.1 mm to ensure strong radiation performance and higher gain. The opening width, W, at the end of the exponential taper section is set to 0.65 mm, which determines the low-frequency cutoff and is constrained by the overall antenna size. The high-frequency cutoff depends on the width at the taper’s starting end and is optimized to balance impedance matching.

**Figure 12:**
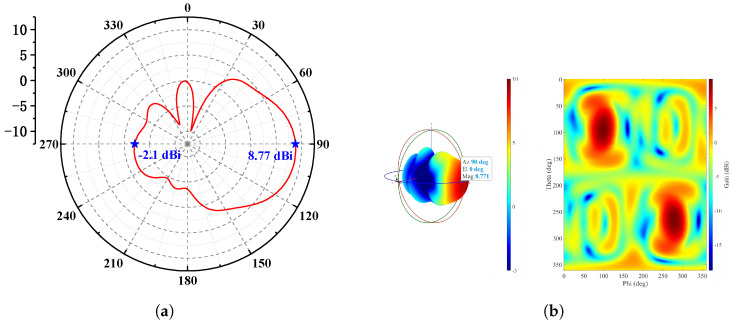
(**a**) E-plane radiation pattern and (**b**) 3D radiation pattern of the Vivaldi antenna at 285 GHz.

To ensure a wide bandwidth, the relative dielectric constant of the substrate should be moderate, and its thickness should not exceed one-tenth of the wavelength (i.e., ≤0.11 mm) to minimize surface waves and parasitic radiation. The metal layer thickness is 2 μm. After full-wave simulation and optimization, the antenna achieves a backlobe gain of −2.1 dBi and a peak gain of 8.77 dBi in the main radiation direction. The E-plane radiation pattern and the 3D radiation pattern are shown in Figure 12a and Figure 12b, respectively.

### 4.2. Photonic Antenna Performance

**Figure 13:**
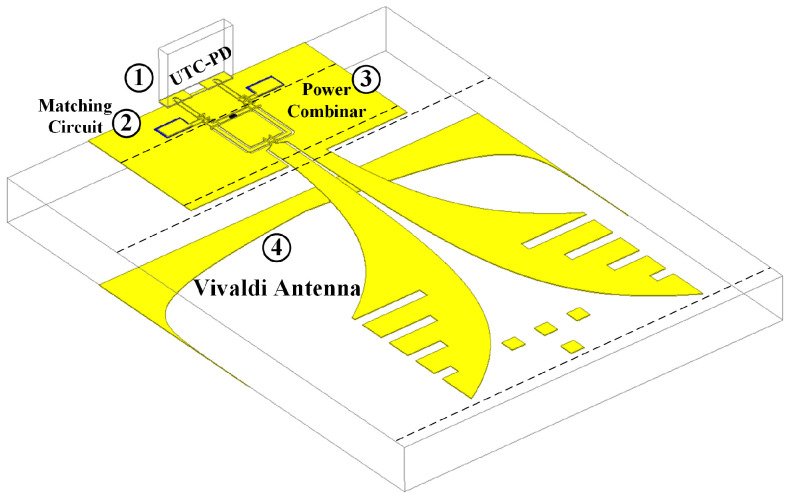
3D structure of the photonic terahertz antenna module.

**Figure 14:**
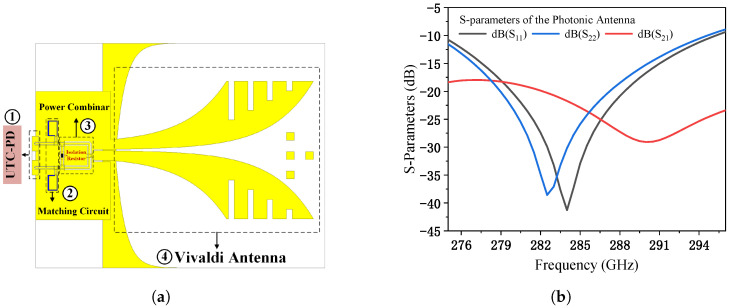
(**a**) Top view of the photonic antenna structure; (**b**) simulated S-parameters.

Figure 13 shows the 3D structure of the photonic antenna. Figure 14a presents the top view of the photonic antenna, while Figure 14b shows the S-parameter curves of the antenna. The MUTC-PD body of the photonic antenna absorbs optical signals to generate electrical signals. The gradient-doped absorption layer of the MUTC-PD introduces a built-in electric field, accelerating the movement of electrons within the absorption layer and enhancing the photoelectric conversion efficiency. The cliff layer between the absorption layer and the collection layer strengthens the electric field in the absorption layer while reducing the electric field in the collection layer, thereby promoting the flow of electrons in the collection region. The signal output from the PD passes through an impedance matching circuit, ensuring that the output impedance of the MUTC-PD is matched to 50 Ω in the 275–296 GHz frequency range, thereby maximizing power transfer and minimizing reflection loss.

Subsequently, the Wilkinson power combiner combines the output signals from two MUTC-PD sources (each with an impedance matching circuit) at the feed port of the Vivaldi antenna, enhancing the overall system output power. Finally, the signal is radiated by the Vivaldi antenna in the form of electromagnetic waves within the 275–296 GHz frequency range, achieving efficient signal transmission.

**Figure 15:**
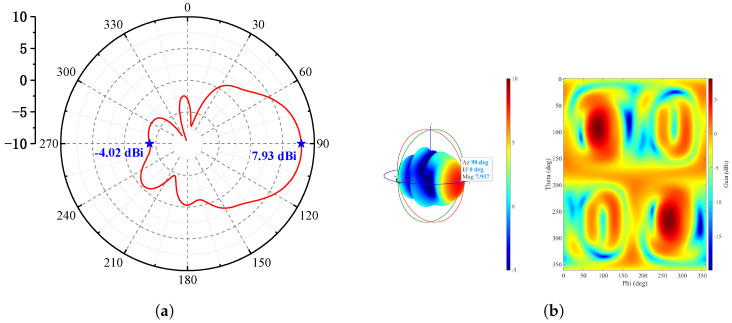
(**a**) E-plane radiation pattern and (**b**) 3D radiation pattern of the complete photonic antenna system at 285 GHz.

Simulation results show that, within the 275–296 GHz frequency band, the isolation between the two input ports of the photonic antenna is approximately −20 dB, and the reflection coefficient at the ports is below −10 dB. At 285 GHz, the antenna gain reaches 7.93 dBi. The E-plane and 3D radiation patterns of the photonic antenna are shown in Figure 15a and Figure 15b, respectively.

## 5. Device Fabrication and Characterization

It should be clarified that, at the current stage, only the baseline MUTC-PD prototype has been fabricated and characterized to verify the device-level modeling and identify the physical bottlenecks limiting high-frequency performance. The full integrated photonic terahertz antenna system is validated through full-wave simulations informed by these experimental findings.

The fabrication process detailed below pertains to the baseline MUTC-PD, which was characterized to identify the performance limitations outlined in the Introduction. It is important to note that these fabricated devices do not correspond to the optimized epitaxial structure presented in the simulated antenna design; rather, their experimental analysis provided the critical insights that informed the subsequent device optimization. For clarity, only the absorption and collection layer thicknesses were modified in the optimized design; all other structural and material parameters remained identical between the baseline and optimized versions.

The devices were fabricated on InP-based wafers using photolithography, metal deposition, and etching. After cleaning and adhesion baking, P-type electrodes were formed with Ti/Pt/Au (20/20/200 nm) by image reversal lithography and evaporation, followed by lift-off and rapid thermal annealing at 350 °C in N_2_ for improved contact. The mesa structure was defined through two-step dry etching and selective wet etching in HCl:H_3_PO_4_, while N-type contacts were made with Ni/Au (20/100 nm) deposition. Device isolation was achieved with mesa etching to a depth of 13 μm. A SiO_2_ dielectric layer (800 nm) was deposited by PECVD, and via holes were created using two lithography and etch cycles. A Ti/Au (300 Å/800 Å) seed layer was sputtered, followed by gold electroplating for 9.5 min, resulting in ∼1.6 μm thick gold with good coverage. After seed layer removal, the Au electrodes were smooth and uniform (1.65 ± 0.2 μm), confirming reliable fabrication and suitable quality for further characterization.

### Device Performance

**Figure 16:**
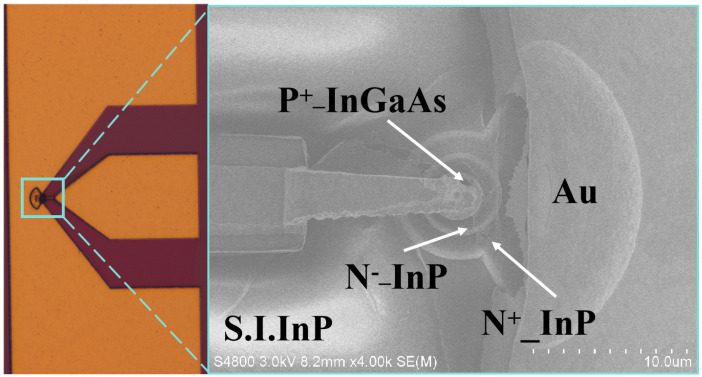
Optical microscope image of the fabricated MUTC-PD. The notations *N*^+^-InP and *N*^−^-InP refer to heavily doped (2 × 10^18^ cm^−3^) and lightly doped (1 × 10^16^ cm^−3^) n-type Indium Phosphide layers, respectively, used for ohmic contacts and electric field management.

**Figure 17:**
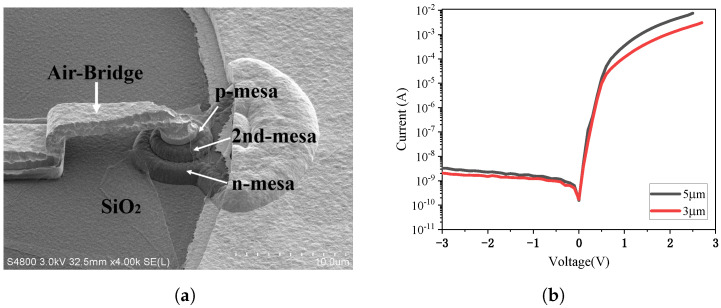
(**a**) Scanning electron microscope (SEM) image of the fabricated MUTC-PD and (**b**) the measured I–V characteristics of devices with 3 μm and 5 μm P-mesa diameters.

Figure 16 presents the optical microscope image of a fabricated MUTC-PD with a 3 μm P-mesa diameter. Figure 17a shows the scanning electron microscope (SEM) image of the fabricated MUTC-PD, where the P-mesa, second mesa, N-mesa, and air-bridge structures can be clearly observed, while Figure 17b shows the measured I–V characteristics of devices with 3 μm and 5 μm P-mesa diameters. Under a reverse bias of −3 V, the dark currents of the 3 μm and 5 μm devices are 2 nA and 3.2 nA, respectively.

The relative frequency response of the device was characterized using a standard optical heterodyne setup. A 1.55 μm continuous-wave laser was amplified by an EDFA and modulated by a Mach–Zehnder modulator driven by an RF signal from a vector network analyzer (VNA). The modulated optical signal was again amplified and directed onto the photodiode under test. The generated electrical signal was separated by a bias-T into DC and RF components, with the RF output fed back to the VNA. By sweeping the RF frequency, the VNA recorded the amplitude and phase response of the photodiode, from which the frequency response curve was obtained.

**Figure 18:**
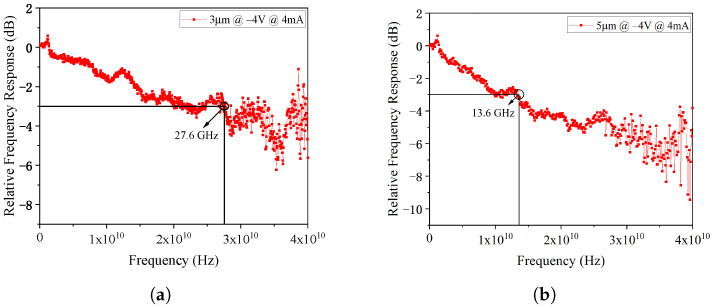
(**a**) The frequency response tested under a fixed reverse bias of 4 V for 3 μm-diameter PD and (**b**) 5 μm-diameter PD.

**Figure 19:**
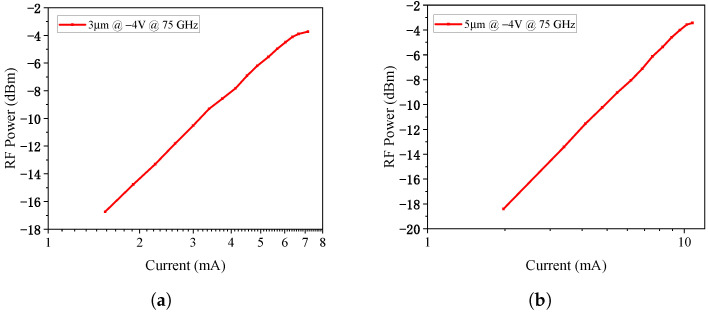
(**a**) The saturation characteristics at −4 V for 3 μm-diameter PD and (**b**) 5 μm-diameter PD.

The frequency response tested under a fixed reverse bias of 4 V are plotted in Figure 18. The 3 μm diameter PD exhibits a 3dB bandwidth of 27.6 GHz at a 4 mA photocurrent, while the 5 μm diameter PD shows a 3 dB bandwidth of 13.6 GHz at the same photocurrent. The saturation characteristics at −4 V bias are shown in Figure 19 at 75 GHz. The 1-dB compression points for the 3 μm and 5 μm PDs occur at photocurrents of 7.15 mA and 10.75 mA, respectively, with corresponding RF saturation powers of −3.73 dBm and −3.44 dBm. These values show significant deviation from the simulation results, which will be analyzed in detail in Section 6.

## 6. Discussion

In this experiment, the epitaxial structure used differs slightly from the design illustrated in Figure 1b. Specifically, the depletion layer thickness is 130 nm and the absorption layer thickness is 150 nm; detailed epitaxial parameters can be found in our previous publication [1]. The epitaxial layers were grown by metal–organic chemical vapor deposition (MOCVD). One known issue with MOCVD growth is dopant diffusion from highly doped regions into adjacent low-doped regions by approximately 100 nm, which leads to a significant deviation between the actual and the ideal epitaxial profiles. This deviation was further confirmed by electrochemical capacitance–voltage (ECV) measurements.

**Figure 20:**
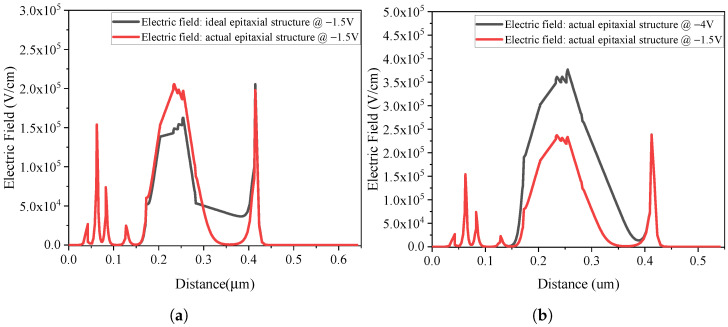
(**a**) Electric field distributions of the actual and ideal epitaxial structures under a reverse bias of 1.5 V. (**b**) Electric field distributions of the actual epitaxial structure under 1.5 V and 4 V.

To evaluate its impact, we simulated and compared the electric field distributions of the actual and ideal epitaxial structures under a reverse bias of 1.5 V, as shown in Figure 20a. The results show that when the doping concentration in the depletion region increases by one order of magnitude (from 1 × 10^16^ to 1.5 × 10^17^/cm^−3^), the electric field decreases correspondingly, with the minimum value approaching zero under the same bias condition.

Experimental characterization revealed that the device bandwidth increased as the reverse bias rose from 1.5 V to 4 V. Under ideal conditions, the electric field at 1.5 V should lie within the optimal range for electron velocity overshoot. However, due to the epitaxial deviation introduced during fabrication, the electric field varies drastically from nearly 0 to over 10 kV/cm as the bias increases, as shown in Figure 20b. Consequently, the velocity overshoot effect is suppressed, reducing the average electron drift velocity from the ideal 3 × 10^7^ cm/s to 2 × 10^7^ cm/s. According to the transit-time model and simulation methods validated in our previous study [1], this reduction in velocity specifically constrains the carrier transit-time-limited bandwidth, resulting in the observed degradation from 325 GHz to 252 GHz.

**Figure 21:**
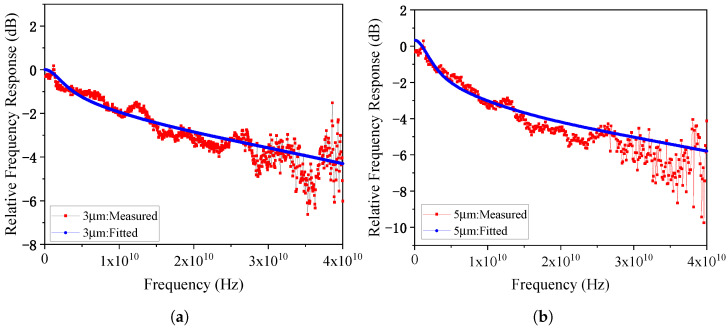
(**a**) The measured and fitted $S_{21}$ parameters for PD with diameter of 3 μm and (**b**) 5 μm under 4 V reverse bias.

In addition to transit-time effects, the RC time constant also contributes to bandwidth degradation, primarily due to high contact resistance. The contact resistance is highly sensitive to annealing conditions such as temperature and duration. After rapid thermal annealing, the specific contact resistivity measured by the CTL-M method was 9.48 × 10^−6^ Ω · cm^2^. Based on this value, the contact resistances for photodiodes with 3 μm and 5 μm P-mesa diameters were calculated to be 301 Ω and 134 Ω, respectively. Incorporating these parameters into the equivalent circuit model shown in Figure 4b, the simulated frequency responses matched well with the experimental results as shown in Figure 21, confirming that suboptimal annealing parameters led to excessive contact resistance, which in turn decreased the RC-limited bandwidth and ultimately reduced the device’s 3 dB bandwidth. These insights provide valuable feedback for epitaxial process control and device-level integration in future photonic THz systems.

To further evaluate the performance of the proposed Photonic Terahertz Generator-A (PTG-A), a comprehensive comparison with other recently reported photonic THz radiation sources is summarized in Table 3. As discussed in the introduction, various strategies have been employed to enhance THz output. For instance, Song et al. utilized a T-junction combiner in a waveguide module to achieve 1.2 mW at 300 GHz [16]. Ohara et al. reported a significant output power of 2.53 mW by focusing on thermal management through the heterogeneous integration of UTC-PDs on SiC substrates [18].

As shown in Table 3, the proposed PTG-A achieves a competitive saturated power of 1.58 dBm (1.44 mW) at 280 GHz. This represents a 4.13× power enhancement compared to a standalone device, validating the effectiveness of the integrated Wilkinson power combiner and impedance matching networks. Our design provides superior port isolation and robust reflection performance without the specialized fabrication complexity required for SiC bonding.

## 7. Conclusions

We present a photonic THz antenna for the 275–296 GHz band, featuring an MUTC-PD optimized based on experimental findings. The simulated design integrates the improved PD with matching networks, a Wilkinson combiner, and a Vivaldi antenna on quartz. It achieves 1.58 dBm saturated output at 280 GHz with 4.13× power enhancement, 7.93 dBi peak gain, and reflection coefficients below –10 dB across the band.

Experiments on earlier photodiodes revealed two key limitations: epitaxial deviations reduced transit-time bandwidth from 325 GHz to 252 GHz, while elevated contact resistance from annealing constraints restricted RC-limited bandwidth. These results guided the MUTC-PD redesign, focusing on depletion layer thickness and contact optimization. Future work will focus on the full-system fabrication and experimental validation. To bridge the gap toward practical implementation, we will employ high-precision Electron-Beam Lithography (EBL) and AuSn flip-chip bonding to minimize parasitic effects and phase imbalances in the combining network. A quasi-optical (QO) measurement platform will be established to prioritize the characterization of Effective Isotropic Radiated Power (EIRP) and far-field radiation patterns. This comprehensive roadmap ensures that the proposed architecture serves as a robust and scalable path toward high-power integrated THz transmitters for next-generation 6G wireless systems.

## Figures and Tables

**Table 1:** Equivalent circuit parameters of the MUTC-PD.

Parameters	Physical Meaning	Value
$R_{InP}$	Junction resistance of InP	311 Ω
$R_{InGaAsP}$	Junction resistance of InGaAsP	228 Ω
$R_{InGaAs}$	Junction resistance of InGaAs	5.5 × 10^4^ Ω
$C_{InP}$	Junction capacitance of InP	2.61 fF
$C_{InGaAsP}$	Junction capacitance of InGaAsP	33.2 fF
$C_{InGaAs}$	Junction capacitance of InGaAs	13.6 fF

**Table 2:** Detailed dimensional parameters of PTG-A.

Parameter	$L_{1}$	$L_{2}$	$L_{3}$	$L_{4}$	Lr	Wr
Value	68 µm	38 µm	66 µm	67.5 µm	12 µm	3 µm

**Table 3:** Performance comparison of state-of-the-art photonic THz radiation sources.

Ref.	Technology	Freq. (GHz)	Power (mW)	Key Features
[16]	2 × UTC-PD + T-junction	300	1.2	Waveguide module
[18]	UTC-PD on SiC	300	2.53	Thermal management
[20]	4 × 1 UTC-PD Array	300	N/A	T-junction array
[17]	MUTC-PD + Vivaldi	100	3.16	W-band integration
This Work	2 × MUTC-PD + Wilkinson	280	1.44	Integrated Antenna

## Data Availability

Data underlying the results presented in this paper are not publicly available at this time but may be obtained from the authors upon reasonable request.
